# Infusion-rate-dependent Acute Neuropathic Pain with Duopa in a Patient with Parkinson's Disease and Pre-existing Neuropathy

**DOI:** 10.7759/cureus.3055

**Published:** 2018-07-26

**Authors:** Harleen Kaur, Kunal Bhatia, Anudeep Yelam, Anukrati Shukla, Irving Asher, Raghav Govindarajan

**Affiliations:** 1 Neurology, Univeristy of Missouri, Columbia, USA; 2 Neurology, University of Missouri, Columbia, USA

**Keywords:** infusion dependent, duopatm, parkinson’s disease, peripheral neuropathy, neuropathic pain

## Abstract

The association of peripheral neuropathy (PN) with the cumulative use of levodopa, presumably by vitamin B12 deficiency, has been well-documented in the literature. However, less is known about the L-dopa infusion-rate-dependent precipitation of pre-existing peripheral neuropathy. We report an unusual case of Parkinson’s disease in a patient who presented with acute exacerbations of pre-existing peripheral neuropathy on exceeding certain rates of continuous Carbidopa-Levodopa Infusions (CLI). We present a case of a 68-year-old gentleman with a 20-year history of idiopathic Parkinson's disease with bilateral subthalamic nucleus deep brain stimulation who was started on Duopa therapy for worsening dyskinesias. Workup before initiating Duopa was significant for idiopathic sensorimotor axonal polyneuropathy, although the symptoms were well controlled with medications. Subsequently, the patient developed severe neuropathic pain within 24 hours of initiating Duopa characterized by burning and stinging in his feet. Symptoms resolved within four hours of reducing the continuous infusion rates without modifying the bolus doses. The patient was doing well with extra bolus doses to manage off periods with no further recurrence of symptoms. With the support of this case report, we would like to conclude that the acute worsening of neuropathic pain with infusion rate or duration of treatment might limit the clinical benefits of Duopa and adds to the expanding spectrum of neurotoxic side effects associated with this therapy. Further prospective studies are needed to monitor the acute worsening of neuropathic pain in patients with pre-existing PN, after initiating CLI and its association with doses of levodopa/carbidopa.

## Introduction

Parkinson's disease (PD) is a neurodegenerative disorder characterized by the progressive degeneration and loss of dopaminergic neurons in the central nervous system (CNS), producing both motor and non-motor symptoms. Replacement therapy with oral levodopa-carbidopa has been the most effective treatment option for improving the motor symptoms of PD [1]. However, in recent years, the continuous intraduodenal infusion of levodopa/carbidopa intestinal gel (LCIG) has demonstrated significant improvement in both motor and non-motor symptoms in advanced PD patients, unamenable to traditional oral drug therapies. In addition to the device/procedure-related adverse effects commonly seen with LCIG therapy, systemic side effects are common and are comparable to oral levodopa-carbidopa therapy [2-3]. However, possible concern has been the development of peripheral neuropathy (PN) in patients receiving LCIG. The most common pattern of involvement noted has been sensory-motor neuropathy with both axonal and demyelinating components, with a variable onset that ranges from subacute to chronic [4-7]. Although the association of peripheral neuropathy with a cumulative dose of oral LCIG is well documented, infusion-rate-dependent precipitation of acute neuropathic pain is an uncommon phenomenon with LCIG therapy. Here, we report an unusual case of Parkinson's disease (PD) with pre-existing neuropathy who developed a rate-dependent exacerbation of neuropathic pain on continuous carbidopa-levodopa infusions (CLI).

## Case presentation

A 68-year-old gentleman with a 20-year history of idiopathic Parkinson’s disease with bilateral subthalamic nucleus deep brain stimulation (left 2-, 1+ amplitude=2.0mV, pulse width=60 microseconds, frequency=130Hz, right 11-, C+ amplitude=1.0mV, pulse width=60 microseconds, frequency=130Hz) was evaluated for Duopa due to worsening dyskinesia. His medications included amantadine 100 mg three times a day and carbidopa-levodopa four to six tablets crushed and infused three times a day over four hours through a G-tube. He had a history of idiopathic sensorimotor axonal polyneuropathy (baseline pain in his feet 3/10 on a visual analog scale). A nerve conduction study done two years ago showed right sural amplitude 2.5 uV, left sural amplitude 2.0 uV (normal > 5 uV), radial sensory 38.0 uV (normal > 15uV), peroneal motor 2.0 mV (normal > 2.5 mV). Neuropathic pain was managed with 300 mg gabapentin once daily. Work-up for neuropathy at that time included vitamin B12 levels, which was 600 pg/ml (Mayo Clinic normal values 200-900 pg/ml), folic acid 10 ng/ml (Mayo Clinic normal 2-20 ng/ml), methylmalonic acid 0.2 nmol/ml (Mayo Clinic <= 0.4nmol/ml), and homocysteine 4 mcmol/L (Mayo Clinic < 13 mcmol/L). The hepatitis profile was negative, HbA1c was 5.5%, serum protein electrophoresis did not show an M spike (monoclonal spikes). The thyroid profile, liver function, fluorescent antinuclear antibody (FANA), and renal functions were also normal. A repeat vitamin B12 and folic acid levels at the time of starting therapy were also normal. The patient was taking vitamin B12 and folic acid supplementation at the time when Duopa was initiated.

At the initial visit, Duopa pump settings were: lock level 1, continuous infusion rate 3.0 cc/h, lockout two hours, extra dose 1.5 ccs, and morning dose nine ccs. The patient developed severe neuropathic pain within 24 hours of initiating Duopa characterized by burning and stinging in his feet (10/10 on a visual analog scale). He had no symptoms after he had morning bolus but four hours after starting the maintenance dose, these symptoms started acutely. The neurological exam was unchanged from before (baseline: absent ankle reflexes and sensory gradient to pinprick at mid-shin level). The continuous rate was reduced to 2.5 cc/h, which did not relieve the symptoms. The gabapentin dose was increased to 600 mg two times a day, and he developed severe dizziness. Duloxetine at 30 mg two times a day made him irritable. Topical lidocaine cream (5%) did not provide any relief. Hydrocodone-acetaminophen 5/325 mg caused him nausea. The continuous infusion rate was brought down to 1.4 cc/h (with series of titrations). The neuropathic pain started to improve (4/10 on a visual analog scale) four hours after reducing the dose to 1.4 cc/hour. He has been using extra dose boluses to manage off periods. The boluses have not resulted in the development of acute neuropathic pain.

## Discussion

In the study by Merola et al., 9% of patients (3/33) were diagnosed with sensory-motor symptomatic PN and 21% (7/33) with subclinical PN on electrophysiological testing at baseline, before initiating LCIG therapy [8]. All of the three patients with symptomatic PN were noted to have mild worsening on repeat electrophysiological testing, and one patient with subclinical PN developed symptomatic sensory axonal neuropathy on LCIG therapy, which was treated with vitamin B12 and folate supplementation. Santos-García et al., in their study, also described a patient that developed axonal neuropathy on LCIG therapy in six months, which improved after LCIG dose reduction and vitamin supplementation [9]. In contrast, our patient developed acute onset, severe neuropathic pain associated with an increase in the rate of CLI despite having a normal serum vitamin B12 and folate levels at baseline.

This case report adds to the expanding spectrum of neurotoxic side effects associated with carbidopa-levodopa infusions. One possible mechanism of LCIG-related PN, mentioned in previous studies, has been a higher bioavailability of L-dopa with CLI infusion compared to oral administration for the same equivalent daily dose, thus contributing to a decrease in vitamin B12 levels possibly by vitamin adsorption deﬁciency or immune-mediated responses, causing subsequent PN [7,10-11]. Several studies have also found a correlation between PN and a high dose of levodopa, prolonged duration of levodopa use, age of patient, low vitamin B12, and high homocysteine and methylmalonic acid levels [4,12-17]. Elevated serum homocysteine levels have been hypothesized to affect neurological function via oxidative stress, mitochondrial dysfunction, inflammation, loss of DNA repair systems, and glutamatergic excitotoxicity [12-14]. An inflammatory-immune imbalance mechanism has also been mentioned in cases developing acute and subacute forms of PN on LCIG therapy [10].

In our patient, a possible explanation for developing acute onset, severe neuropathic pain after initiating CLI could be the sharp rise in homocysteine levels after higher doses of levodopa were delivered intestinally, causing oxidative stress and acute inflammation. However, the presence of an inflammatory-immune mechanism cannot be entirely ruled out. Based on the existing data and the present clinical scenario, we recommend that neurologists should be cautious of the possible development of acute onset, severe neuropathic pain in patients with pre-existing PN after initiating CLI. Further prospective studies are needed to monitor the acute worsening of neuropathic pain in patients with pre-existing PN after starting CLI and elucidate its association with doses of levodopa/carbidopa and the duration of treatment.

## Conclusions

An acute worsening of pre-existing neuropathic pain with a higher infusion rate or a longer duration of treatment might limit the clinical benefits of Duopa and adds to the expanding spectrum of neurotoxic side effects associated with this therapy. Further prospective studies are needed to monitor the acute worsening of neuropathic pain in patients with pre-existing PN after initiating CLI and to understand its association with doses of levodopa-carbidopa.
